# The role of GLI-SOX2 signaling axis for gemcitabine resistance in pancreatic cancer

**DOI:** 10.1038/s41388-018-0553-0

**Published:** 2018-10-31

**Authors:** Yanfei Jia, Dongsheng Gu, Jun Wan, Beiqin Yu, Xiaoli Zhang, E. Gabriela Chiorean, Yunshan Wang, Jingwu Xie

**Affiliations:** 1 Central Laboratory, Jinan Central Hospital Affiliated to Shandong University, Jinan, China; 2 Wells Center for Pediatric Research, Department of Pediatrics, Indiana University school of Medicine, Indianapolis, IN 46202 USA; 3 Department of Molecular and Medical Genetics, Indiana University Simon Cancer Center, Indiana University, Indianapolis, IN 46202 USA; 4 Shanghai Key Laboratory of Gastric Neoplasms, Shanghai Institute of Digestive Surgery, Ruijin Hospital, Shanghai Jiao Tong University School of Medicine, Shanghai, 200025 China; 5 Division of Medical Oncology, University of Washington, and Fred Hutchinson Cancer Research Center, 825 Eastlake Ave E, G4-833, Seattle, WA 98109-1023 USA

**Keywords:** Cancer therapeutic resistance, Prognostic markers

## Abstract

Pancreatic cancer, mostly pancreatic ductal adenocarcinomas (PDAC), is one of the most lethal cancers, with a dismal median survival around 8 months. PDAC is notoriously resistant to chemotherapy. Thus far, numerous attempts using novel targeted therapies and immunotherapies yielded limited clinical benefits for pancreatic cancer patients. It is hoped that delineating the molecular mechanisms underlying drug resistance in pancreatic cancer may provide novel therapeutic options. Using acquired gemcitabine resistant pancreatic cell lines, we revealed an important role of the GLI-SOX2 signaling axis for regulation of gemcitabine sensitivity in vitro and in animal models. Down-regulation of *GLI* transcriptional factors (*GLI1* or *GLI2*), but not SMO signaling inhibition, reduces tumor sphere formation, a characteristics of tumor initiating cell (TIC). Down-regulation of *GLI* transcription factors also decreased expression of TIC marker CD24. Similarly, high *SOX2* expression is associated with gemcitabine resistance whereas down-regulation of *SOX2* sensitizes pancreatic cancer cells to gemcitabine treatment. We further revealed that elevated *SOX2* expression is associated with an increase in *GLI1* or *GLI2* expression. Our ChIP assay revealed that GLI proteins are associated with a putative Gli binding site within the *SOX2* promoter, suggesting a more direct regulation of *SOX2* by GLI transcription factors. The relevance of our findings to human disease was revealed in human cancer specimens. We found that high SOX2 protein expression is associated with frequent tumor relapse and poor survival in stage II PDAC patients (all of them underwent gemcitabine treatment), indicating that reduced *SOX2* expression or down-regulation of *GLI* transcription factors may be effective in sensitizing pancreatic cancer cells to gemcitabine treatment.

## Introduction

The overall survival of cancer patients has significantly improved in the last decade due to the use of multidisciplinary care, improved chemotherapeutic agents, development of novel targeted biologic agents in combination of cancer genomic profiles and improved palliative care services [1]. In contrast, the overall survival of pancreatic cancer patients, particularly patients with pancreatic ductal adenocarcinomas (PDAC), has not changed very much in the last 40 years [1–4]. Upon diagnosis, PDAC patients have a dismal median survival around 8 months and ~ 8% 5-year survival rate. Pancreatic cancer is predicted to be number two cancer killer by 2030 [4]. The notorious resistance of pancreatic cancer to the traditional cytotoxic chemotherapeutical agents and targeted therapy poses major challenge in reducing the death toll from this deadly disease.

Gemcitabine has been the first line therapeutic agent for patients with advanced pancreatic cancer since 1997 [3]. Despite initial responsiveness to gemcitabine, pancreatic cancer eventually develops resistance, and patients succumb to the disease. Gemcitabine is a deoxycytidine analog with specific spectrum of activity and several unique properties. Gemcitabine is known to kill cells with active DNA synthesis by blocking the G1/S transition. Combining albumin-bound paclitaxel with gemcitabine has shown clear therapeutic advantage than gemcitabine alone (8.5 vs. 6.7 months in median survival) [5]. The exact molecular mechanisms underlying gemcitabine resistance in pancreatic cancer is not completely understood. Previous study indicates that stromal hedgehog signaling may be responsible for lack of gemcitabine penetration to the tumor in the mouse model. It was thus predicted that inhibition of hedgehog signaling may be effective in promoting gemcitabine efficacy [6]. However, clinical trials combining gemcitabine and hedgehog inhibitor IPI-262 did not benefit the pancreatic cancer patients, and we believe our study explains why the clinical trials failed. Gemcitabine resistance can be either intrinsic or acquired. It has been hoped that strategies to overcome gemcitabine resistance may be effective in prolonging the lifespan of pancreatic cancer patients.

In this study, we analyzed gemcitabine resistant cell lines by comparison with their matched sensitive counterparts. We discovered up-regulation of the GLI-SOX2 signaling axis in the resistant cells, which is consistent with published data that support the role of cancer stem cells is drug resistance [7–9]. We have performed a number of experiments to prove the significance of this signaling axis for gemcitabine resistance both in cultured cells and in animal models. We have detected regulation of *SOX2* by *GLI* transcriptional factors in pancreatic cancer cells. The relevance of our data to pancreatic cancer was reflected by the significant association between a high SOX2 protein level with an increased risk of tumor relapse and a poor survival of pancreatic cancer patients who underwent gemcitabine-based chemotherapy.

## Results

### Molecular characterization of gemcitabine resistant pancreatic cancer cells

**Fig. 1:**
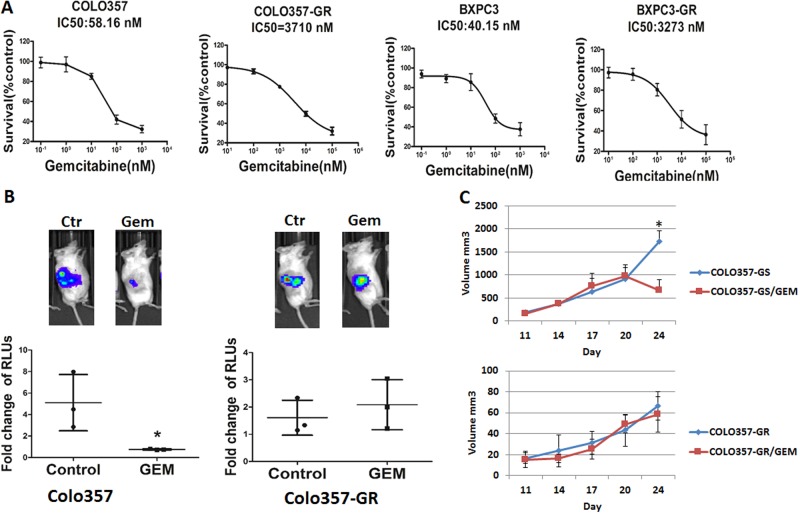
Characterization of gemcitabine resistance of pancreatic cancer cells. **a** shows the IC50 values for gemcitabine in COLO357, COLO357-GR, BXPC3 and BXPC3-GR cells. Data points are average of duplicate wells from two independent assays. **b** shows a different response of COLO357 in comparison with COLO357-GR to gemcitabine in orthotopic pancreatic cancer models. **c** shows the growth curves of subcutaneous tumors following gemcitabine treatment or left untreated (vehicle control). The top shows the tumor growth curve from Colo357 parental cells (shown as Colo357-GS), and the bottom shows the tumor growth curve from Colo357-GR cells (shown as Colo357-GR). Gemcitabine treatment group was shown as GEM. *p value < 0.05 based on Student’s *t* test

To understand the molecular basis of gemcitabine resistance, we first characterized two gemcitabine resistant cell lines established from their corresponding parental cell lines Colo357 and BxPC3 following multiple treatments with gemcitabine. The IC50 for gemcitabine in the resistant Colo357 cells (named as Colo357-GR) is 3710 nM whereas that of the parental cells is only 58.16 nM. The calculated resistant index (RI) [10–12] is ~ 63.8 (=3710/58.16), indicating a significant gemcitabine resistance in Colo357-GR. Similarly, the IC50 for resistant BxPC3 cells (named as BxPC3-GR) is 3273 nM whereas that for the parental cells is 40.15 nM. The RI for BxPC3-GR is also very high (81.5 = 3273/40.15) (Fig. 1a).

We further tested the response of Colo357-GR-derived tumors to gemcitabine treatment in the immune deficient NSG mice following pancreatic injection. Our results showed that gemcitabine (25 mg/kg via tail vein) had no effects on tumors from Colo357-GR cells but significantly reduced the tumors derived from the parental Colo357 cells (Fig. 1b). We also performed subcutaneous injection of Colo357-GR and the parental Colo357 cells, and performed gemcitabine treatment after tumors were formed. We found that the tumors derived Colo357 continued to grow, the tumors derived from the parental Colo357 cells shrunk after gemcitabine treatment (Fig. 1c). The data from both orthotopic and subcutaneous models gave essentially the same result: tumors derived from Colo357-GR cells are indeed gemcitabine resistant in mice. Similarly, we found that tumors from gemcitabine resistant BxPC3-GR cells are not sensitive to gemcitabine in comparison with their parent cells (as BxPC3-GS) (Fig. S1). These data confirm that the tumors derived from these gemcitabine resistant cells do not respond well to gemcitabine treatment.

**Fig. 2:**
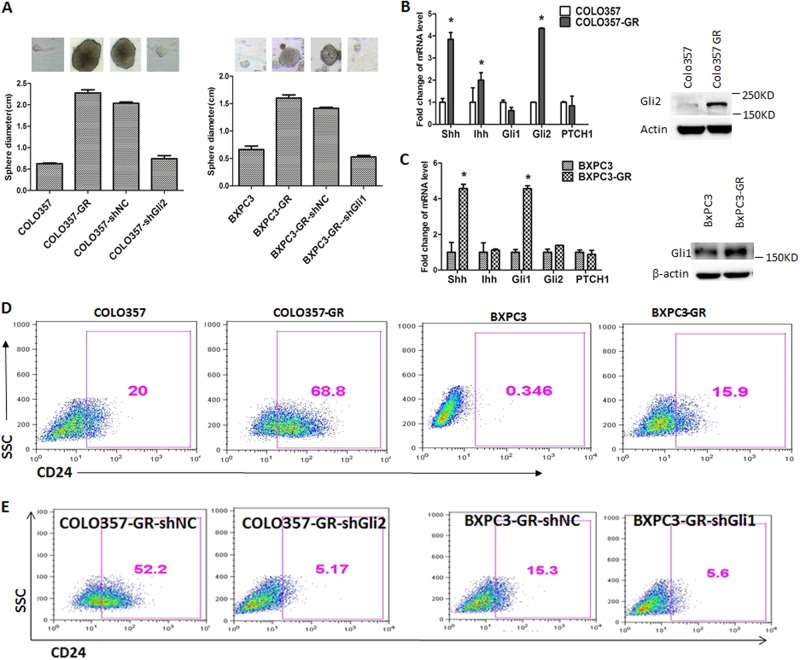
Association of elevated *GLI* expression with tumor sphere formation and CD24 expression. **a** shows a summary of tumor sphere data in gemcitabine resistant Colo357 cells (shown as Colo357-GR) and the parental cells (shown as Colo357) on the left, and gemcitabine resistant BxPC3 (shown as BxPC3-GR) and the parental cells (shown as BxPC3) on the right. The top shows the typical tumor sphere morphology, and the bottom panel shows the average diameter of the tumor spheres. **b** shows the relative expression of Hh pathway molecules in Colo357 cells using quantitative PCR (qPCR). **c** shows the relative expression of Hh pathway molecules in BxPC3 cells using quantitative PCR (qPCR). We also detected GLI1 and GLI2 proteins (shown at the right). **d** shows flow cytometry data of CD24 positivity (percentage) in different cell lines. **e** shows CD24 positivity (as percentage) in difference cell lines after shRNA expression. *p value < 0.05 based on Student’s *t* test

Previous studies indicate that residual cancer cells or the putative tumor initiating cells (TICs) may be responsible for chemo-resistance [13]. Putative TICs are characterized as cells forming tumor sphere efficiently, and are regulated by several signaling pathways involved in embryonic development, such as wnt, hedgehog and notch signaling [14–16]. We compared tumor sphere formation between the resistant Colo357-GR and their matched parental cells, and found that Colo357-GR cells formed large and round spheres whereas the parental cells barely formed any spheres (Fig. 2a left). This phenomenon is not cell line-specific because BxPC-GR cells also formed larger tumor spheres in comparison with the parental BxPC3 cells (Fig. 2a right). This observation suggests the presence of more TICs in the resistant cells.

Next, we compared gene expression in pathways responsible for maintenance of residual cancer cells or tumor initiating cells. Hedgehog, Wnt and Notch signaling pathways play important roles in embryonic development, and are critical for maintenance of putative TICs [14–16]. As shown in Fig. 2b and Fig. 2c, we found that *GLI* molecules (*GLI1* or *GLI2*) were significantly up-regulated in the gemcitabine resistant cells. Tumors formed from Colo357-GR had higher *GLI2* expression than those derived from Colo357 parental cells (see Fig. S2), which is consistent with gene expression in cell lines (Fig. 2b). We confirmed high GLI2 protein expression in the gemcitabine resistant Colo357-GR cells (Fig. 2b right). With specific antibodies to GLI1, we confirmed GLI1 protein expression in BxPC3 and BxPC3-GR cells (Fig. 2c right). In the two pairs of cell lines, we did not detect *GLI1* isoforms *GLI1ΔN* and *tGLI1* [17, 18], suggesting that elevated *GLI1* expression was mainly from the full-length *GLI1*.

In our previous studies, we have shown that hedgehog signaling activation is one of the major underlying mechanisms for chemotherapy resistance in gastric and colorectal cancers [19, 20], indicating that Hh signaling may be responsible for gemcitabine resistance in pancreatic cancer. In contrast, we did not observe significant gene expression changes in *DKK1*, *JAG2* or *CTGF*, molecules involved in Wnt, Notch and Hippo/YAP signaling (see Fig. S3).

To determine whether our data from two independent cell lines are also present in other pancreatic cancer cells, we searched published gene expression data on gemcitabine resistant cell lines from GEO database. The dataset GSE35141 showed high expression of hedgehog signaling molecules or target genes in gemcitabine resistant cells. For examples, *GLI1* and *GLI2* were activated by 3.0-fold (*p* = 6.5E-3) and 2.7-fold (*p* = 1.4E-2), respectively, in resistant PK-1 cells compared to the parental ones. *GLI2* was also expressed at 23-fold (*p* = 1.4E-5) in resistant PK-9 cell line. Thus, it appears that up-regulated hedgehog signaling is a signature for gemcitabine resistance in several pancreatic cancer cell lines.

To further characterize the residual cancer cells or tumor initiating cells, we examined cell surface markers in these cells. Expression of CD24, CD44, and ESA are enriched in pancreatic cancer stem cells [21]. In BxPC3, Colo357 as well as the derived gemcitabine resistant cell lines, CD44 and ESA are highly expressed. We did find more CD24 positive cells in the gemcitabine resistant cells (Colo357-GR > 68%; Colo357 20%; BxPC3-GR~16%; BxPC3~0.3%, *p* = 0.0061) (Fig. 2d), suggesting that gemcitabine resistant cell lines have a high percentage of putative TICs. We also examined expression of several signaling molecules whose expression has been associated with chemo-resistance in other cancer types, such as ABCG2, c-FLIP, BCL2 [22–24]. However, we did not observe significant changes in these genes (see Fig. S4).

The above characterization of two pairs of cell lines allows us to investigate the underlying mechanisms for gemcitabine resistance in pancreatic cancer cells.

### Regulation of the putative TIC population and gemcitabine resistance in pancreatic cancer cells

To directly test the significance of hedgehog signaling for putative TIC maintenance, we detected the putative TIC population in pancreatic cancer cells using two approaches.

First, we measured tumor sphere formation efficiency in Colo357-GR cells with *GLI2* shRNAs (as Colo357-GR-shGli2) or Colo357-GR cells with a scrambled shRNA (as Colo357-GR-shNC). Tumor sphere formation efficiency is a known biological readout of TICs [25]. We found that *GLI2* knockdown significantly reduced the size of tumor spheres (Fig. 2a left). In BxPC3-GR cell line in which *GLI1* is up-regulated, knockdown of *GLI1* reduced the size of tumor spheres (Fig. 2a right). These results indicate that *GLI* transcription factors are required for tumor sphere formation in the gemcitabine resistant cells.

Second, we detected cell surface marker expression following alteration of *GLI2* level in Colo357-GR. We found that *GLI2* knockdown significantly reduced expression of CD24 (Fig. 2e). Similar results were also observed in BxPC3-GR cells (Fig. 2e). These data indicate that reduced Hh signaling decreases expression of putative TIC surface marker CD24. We also examined ALDH + cells and side population, but did not find any difference between the gemcitabine resistant cells and the parental cells (data not shown).

From the above data, we conclude that Hh signaling activation is important for maintenance of the putative TIC population as indicated by tumor sphere formation and expression of putative TIC surface marker CD24.

**Fig. 3:**
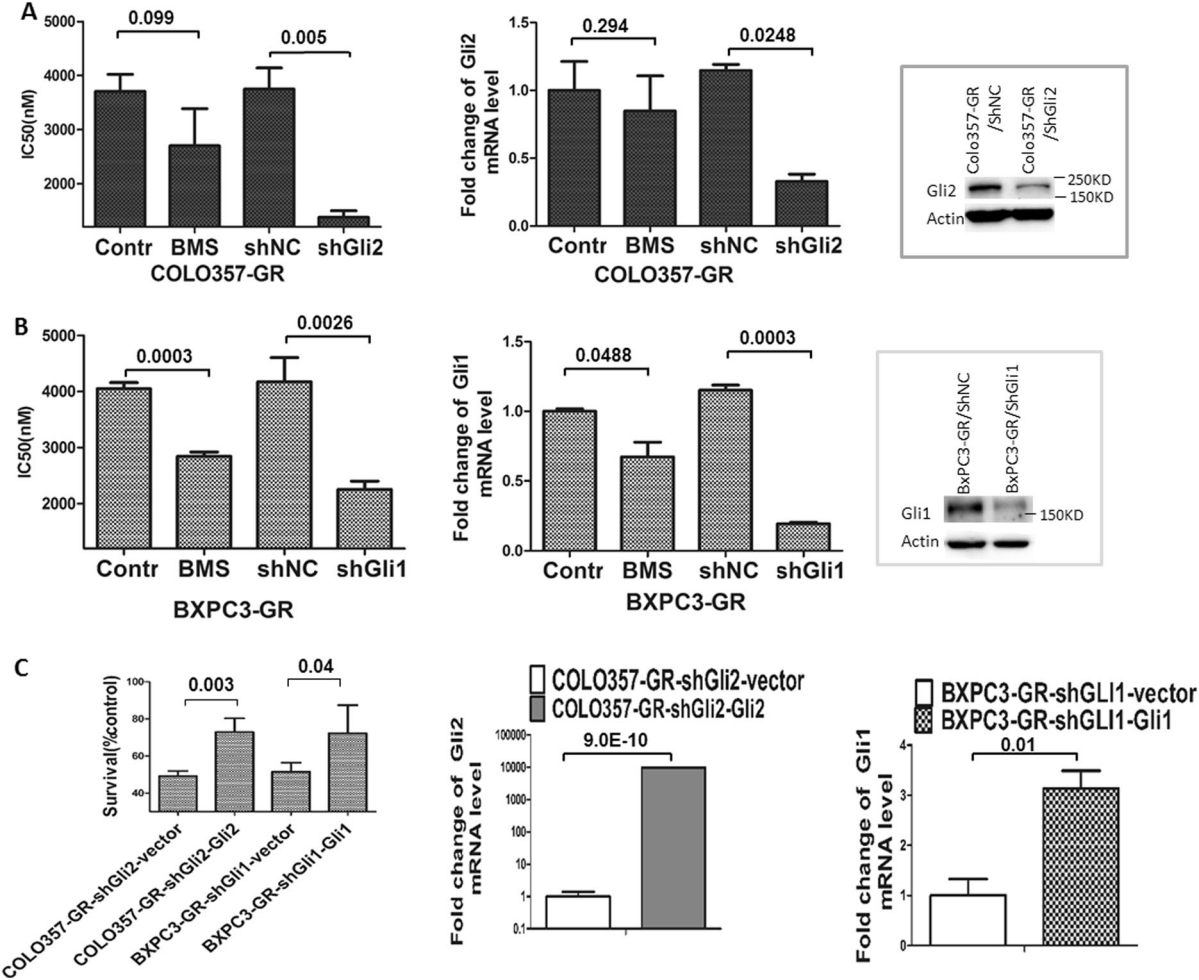
Reduced expression of GLI transcription factors, but not SMO inhibition, sensitizes pancreatic cancer cells to gemcitabine treatment. **a** shows the treated data from SMO antagonist BMS-833923 (shown as BMS in the figure) or *GLI2*-shRNAs in COLO357-GR cells. *GLI2* shRNAs, but not BMS-833923, were more effective in sensitizing COLO357-GR cells to gemcitabine treatment (left). *GLI2* shRNAs reduced expression of *GLI2* while BMS833923 had little effects (right). **b** shows the data from treatment with BMS-833923 or *GLI1*-shRNAs in BXPC3-GR cells. *GLI1* shRNAs, but not BMS833923, were more effective in sensitizing BXPC3-GR cells to gemcitabine treatment (left). The *GLI1* level was reduced more significantly by *GLI1* shRNAs than by BMS-833923 (Center). GLI1 protein was also detected by Western blotting (right). **c** shows the specificity of shRNAs by re-expression of *GLI1* or *GLI2* and their effects on cell viability after gemcitabine (2 mM) treatment. Overexpression of GLI2 or GLI1 increased gemcitabine resistance in COLO357-GR-shGli2 and BXPC3-GR-shGli1 cells (left). Expression of *GLI1* (bottom right) and *GLI2* (bottom left) was detected by real-time PCR. *p* < 0.05 was regarded as significant changes

Furthermore, we determined whether knockdown *GLI1* or *GLI2* affect gemcitabine sensitivity in the resistant cells. When *GLI2* shRNAs were expressed in Colo357-GR, we found a significant decrease in gemcitabine IC50 (Fig. 3a). Similarly, when *GLI1* shRNAs were expressed in BxPC3-GR, the IC50 was also significantly reduced (Fig. 3b).

The specificity of *GLI1* and *GLI2* shRNAs was tested by ectopic expression of *GLI1* in *GLI1*-shRNAs-expressing BxPC3 cells (or ectopic expression of *GLI2* in Colo357-GR-sh*Gli2* cells). We found that ectopic expression of *GLI1* reversed the phenotype of *GLI1* shRNAs in BxPC3-GR cells and became more resistant (Fig. 3c left), and the similar results were also obtained using ectopic *GLI2* expression in Colo357-GR cells (Fig. 3c left). These results indicate the specificity of *GLI1* and *GLI2* shRNAs, and further confirm that elevated expression of *GLI* transcription factors is sufficient to drive gemcitabine resistance.

We noticed elevated expression of *SHH* and *IHH* in the resistant cells, and thought that elevated expression of *GLI1* or *GLI2* in the resistant cells may be through hedgehog ligand-mediated signaling. In that case, Smoothened antagonist BMS833923 [26] should be sufficient to reduce IC50 for gemcitabine. However, our data showed that BMS833923 only reduced gemcitabine IC50 by less than 50% (Fig. 3a, b). Furthermore, BMS833923 was not as effective as *GLI* specific shRNAs in reducing expression of *GLI1* (in BxPC3-GR cells) or *GLI2* (in Colo357-GR cells). These data indicate that ligand-independent (non-canonical) hedgehog signaling plays a more important role in gemcitabine resistance. This result is consistent with the failed clinical trials using Smoothened antagonists [27–29]. Currently, the exact mechanisms responsible for this non-canonical Hh signaling activation are under investigation.

**Fig. 4:**
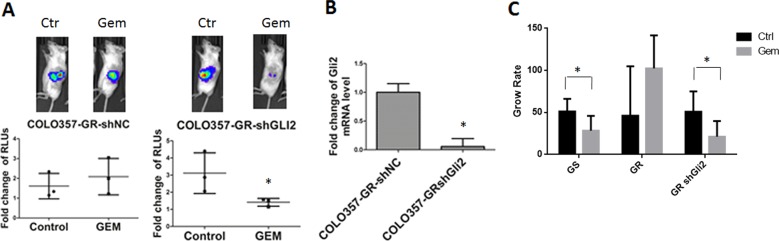
The effect of *GLI2* knockdown on gemcitabine response in an orthotopic mouse model. **a** shows the effect of *GLI2* knockdown in tumor response to gemcitabine in mice (left for the Colo357-GR-shNC as control, right for Colo357-GR-sh*GLI2*). **b** shows down-regulation of Gli2 as confirmed by qPCR analysis. **c** shows the data from subcutaneous tumors (GS as Colo357 parental cells; GR as Colo357-gemcitabine resistant cells; GR-sh*GLI2* as *GLI2* shRNA expressing Colo357-GR cells). Statistically significant findings were denoted when *p < 0.05

To assess whether tumors formed from these cells respond to gemcitabine as expected, we treated tumor-bearing mice with gemcitabine. After tumors develop to certain size following injection of different cancer cells (Colo357-GS; Colo357-GR; Colo357-GR-shNC; Colo357-GR-Gli2-shRNAs), we treated mice with gemcitabine (25 mg/kg, twice weekly). Although Colo357-GS cells formed relative large tumors, the tumors shrank rapidly after gemcitabine treatment. In contrast, Colo357-GR-formed tumors did not respond to gemcitabine treatment as we had expected (Fig. 4a). When *GLI2*shRNAs were expressed in Col357-GR cells, *GLI2* expression was significantly reduced (Fig. 4b), and tumors became sensitive to gemcitabine (Fig. 4A). In contrast, the shRNA control- (Colo357-GR-shNC) formed tumors were not responsive to gemcitabine treatment (Fig. 4a). We have performed two types of studies in immune deficient NSG mice: orthotopic mouse model following pancreatic injection of luciferase-expressing cells [26] or subcutaneous injection of cells (Fig. 4c). Our data from both models indicate that Gli transcription factors are essential factors in mediating gemcitabine resistance in pancreatic cancer.

Similarly, we found that while tumors derived from BxPC3-GR were less sensitive to gemcitabine (Fig. S1), *GLI1* gene knockdown using specific shRNAs sensitized tumors (for the tumors derived from BxPC-GR-sh*GLI1*) to gemcitabine treatment (Figure S5).

Taken together, we found that elevated *GLI1* and GLI2, but not the ligands *SHH* and IHH, is required for the acquired gemcitabine resistance in pancreatic cancer both in cultured cells and in mice. We believe that hedgehog signaling exerts its functions through regulation of the putative TICs.

### Regulation of SOX2 by hedgehog signaling in gemcitabine resistant cells

**Fig. 5:**
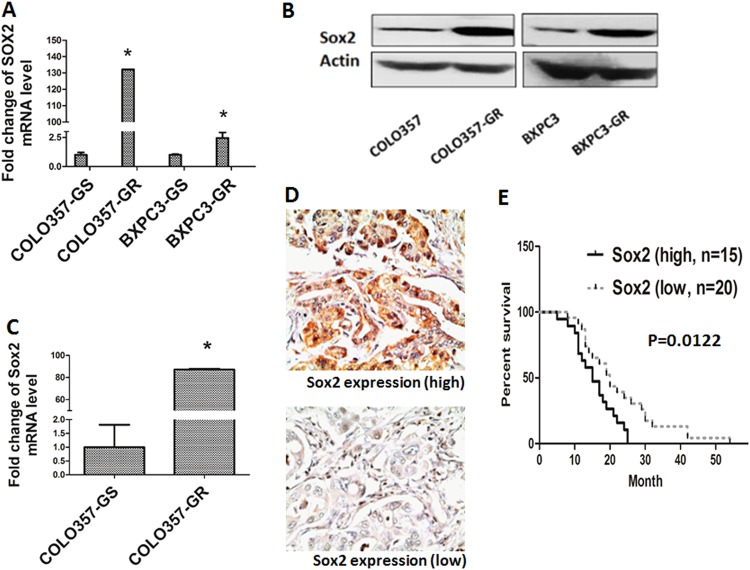
Association of *SOX2* expression with gemcitabine resistance in pancreatic cancer cell lines and patient survival. **a** and **b** show the relative gene (**a**) and protein (**b**) expression of SOX2 in different cell lines. **c** shows the relative transcript level of *SOX2* in the orthotopic mouse models of pancreatic cancer (Colo357-GS as colo357 parental cells and Colo357-GR as gemcitabine resistant Colo357 cells). **d** shows typical high and low SOX2 staining in human pancreatic cancer specimens. **e** shows the Kaplan–Meier plots of patients with high or low *SOX2* expression (Long-rank test). Statistically significant findings were denoted when *p < 0.05

A number of transcription factors have been reported to regulate putative TICs, and we assessed expression of these factors [30–37] in the resistant cells, and found that expression of *SOX2* was highly elevated (Fig. 5a). A significant increase in SOX2 protein level was also observed (Fig. 5b) in the resistant cells.

It is known that sex-determining region Y-box 2 (Sox2) is of vital importance in regulation of stem cells in embryos and in cancer [38]. In the tumors derived from Colo357-GR, we found a higher level of *SOX2* in comparison to the parental COLO357 cells (see Fig. 5c). The relevance of *SOX2* expression to human pancreatic cancer specimens was investigated using a tissue array assembled with surgically removed specimens from stage II pancreatic cancer patients enrolled in our medical center. All the patients had stage II PDAC tumors and underwent chemotherapy with gemcitabine as the first line treatment. This tissue array is suitable for protein detection by immunohistochemistry. We examined SOX2 protein expression by immunohistochemistry (Fig. 5d), and found that some tumors with higher SOX2 protein expression while others express a low level of SOX2. Since all patients had used gemcitabine following surgery, tumor relapse following gemcitabine treatment may result in a short survival of patients. We performed Kaplan-Meier analysis for these patients, and found that patients with a high SOX2 protein expression had poor survival (*p* < 0.05) (Fig. 5e). These results suggest that a high protein expression of SOX2 indicates poor prognosis of gemcitabine treatment and patient survival.

**Fig. 6:**
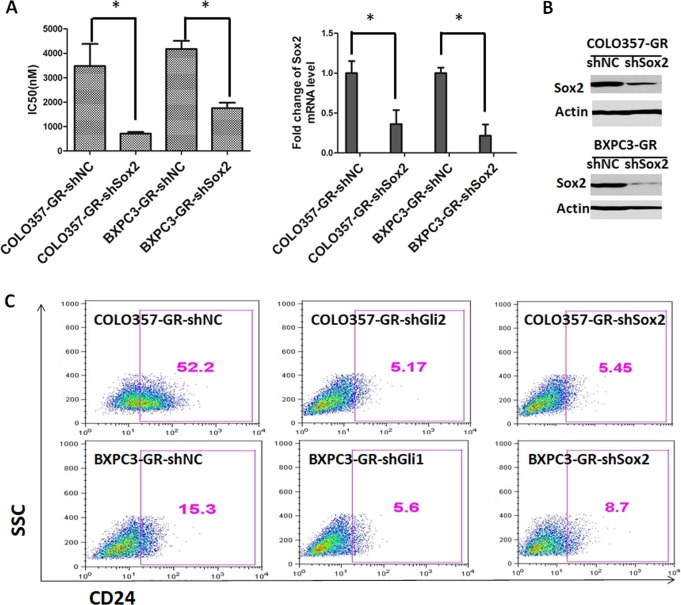
The role of SOX2 in gemcitabine resistance. **a** shows the effect of *SOX2* knockdown on gemcitabine treatment (left shows the IC50 change, right shows the relative gene expression), and **b** shows the SOX2 protein level. **c** shows the effect of shRNAs of *GLI2* (COLO357-GR), *GLI1* (BxPC3-GR) and *SOX2* on CD24 positivity following flow cytometry analysis

To determine the functional relevance of SOX2 expression with gemcitabine sensitivity in pancreatic cancer cells, we knocked down *SOX2* expression by two independent shRNA constructs in the gemcitabine resistant cell lines and treated with gemcitabine afterwards. As indicated in Fig. 6a & 6b, down-regulation of *SOX2* significantly reduced the IC50 of gemcitabine in the resistant cancer cells. For example, Colo357-GR cells with *SOX2*shRNA expression has an IC50 of gemcitabine < 1000 nM in comparison with the IC50 of Colo357-GR with control shRNA above 3000 nM. Similarly, *SOX2*shRNAs reduced the IC50 of BxPC3-GR from over 3000 nM to less than 2000 nM (Fig. 6a, b). *SOX2* shRNAs also reduced the level of CD24 positivity in the resistant cells (Fig. 6c), just like *GLI2* shRNAs in Colo357 cells (Fig. 2e). These results indicate that SOX2 is at least partly responsible for gemcitabine sensitivity.

**Fig. 7:**
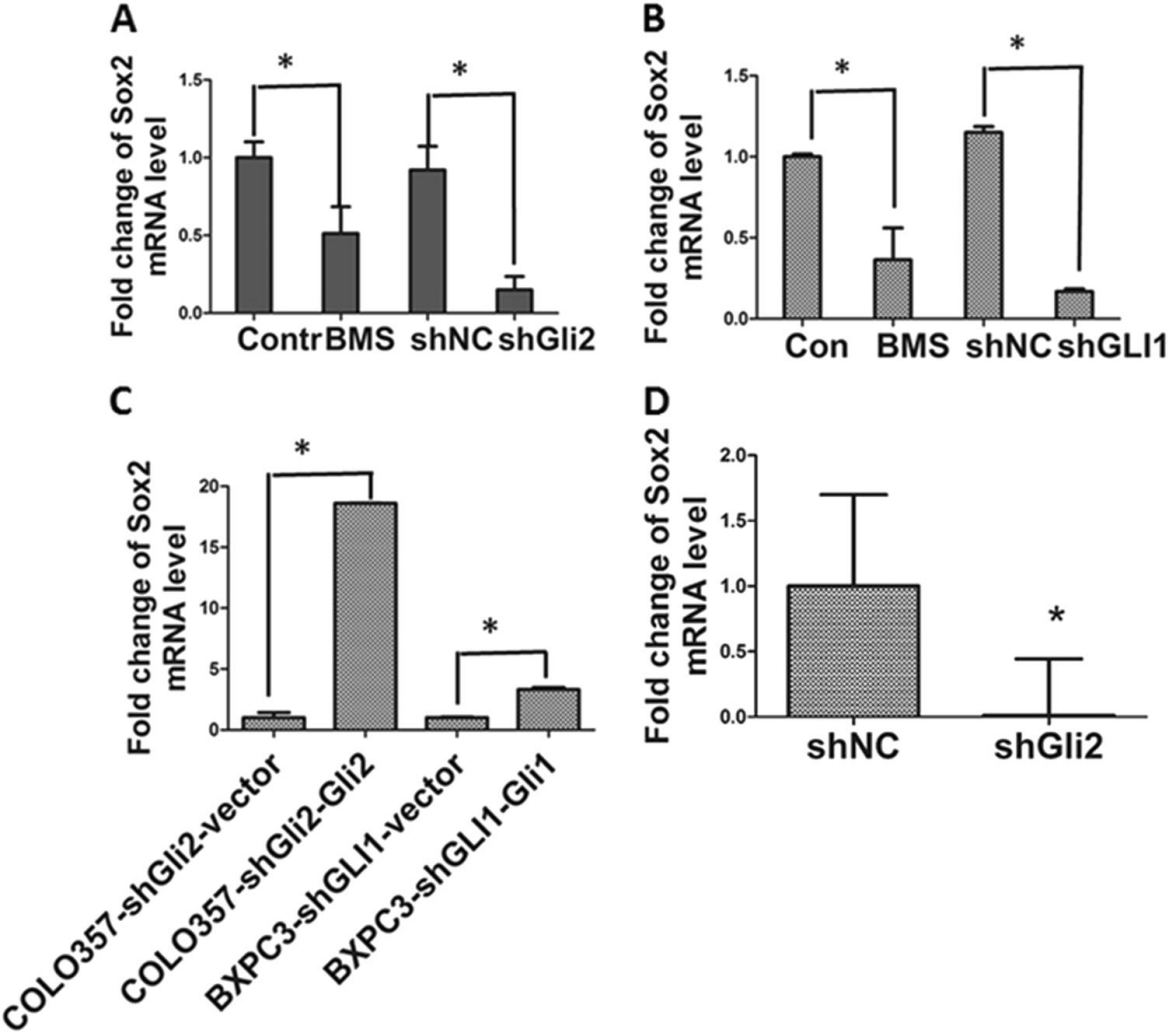
Regulation of *SOX2* by *GLI1* and *GLI2*. **a** shows *SOX2* expression following treatment with BMS-833923, a specific inhibitor for SMO or expression of *GLI2* shRNAs in COLO357-GR cells. **b** shows Sox2 expression following treatment with BMS-833923, a specific inhibitor for SMO or expression of *GLI2* shRNAs in BxPC3-GR cells. **c** shows regulation of *SOX2* by ectopic expression of *GLI1* and *GLI2* in COLO357 and BxPC3 cells (similar to Fig. 3c). **d** shows reduced expression of *SOX2* by *GLI2* shRNAs in tumors derived from COLO357-GR-control shRNA and COLO357-*GLI2*-shRNAs in an orthotopic mouse model

Next, we investigated how *SOX*2 expression was induced in the resistant pancreatic cancer cells. We have evidence to indicate that the level of *GLI1/2* is correlated to *SOX2* expression. First, knockdown of *GLI2* (Fig. 7a) or *GLI1* (Fig. 7b) was associated with reduced expression of *SOX2*. Second, re-expression of *GLI1* or *GLI2* induced *SOX2* expression (Fig. 7c). In the mouse model, tumors formed from Colo357-GR, in which *GLI2* is highly expressed, had more *SOX2* expression in comparison with the tumors from the parental Colo357 cells (Fig. 7d). Thus, it appears that the level of *SOX2* expression is controlled by *GLI1/2* expression.

**Fig. 8:**
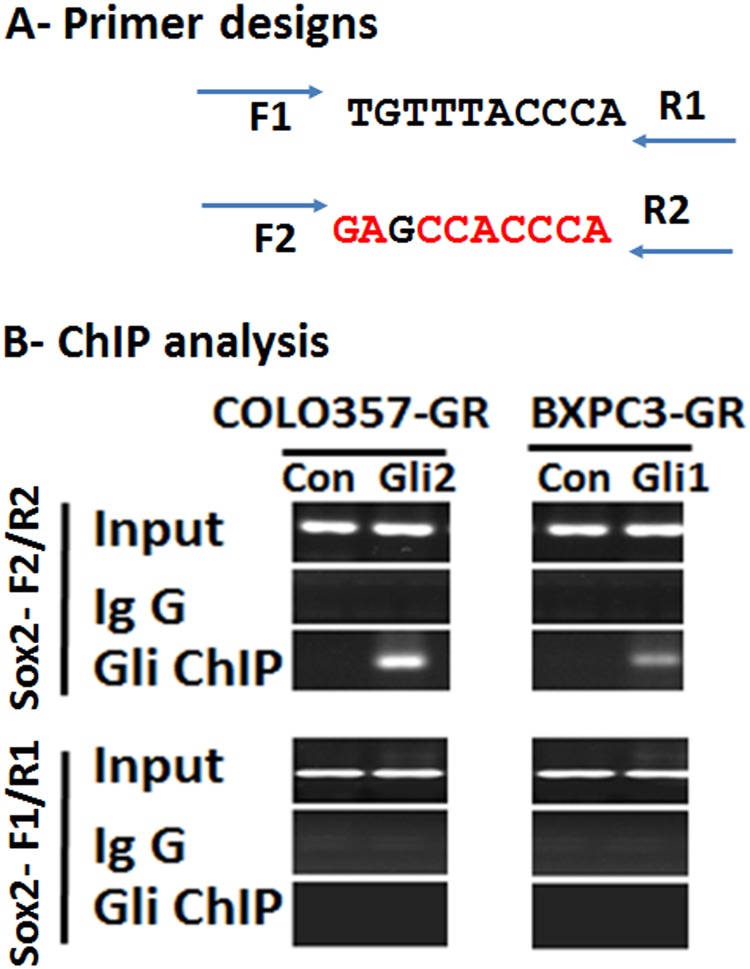
Binding of *SOX2* promoter fragments by *GLI1* and *GLI2* proteins as revealed by ChIP. **a** shows a putative GLI binding site in a PCR product from primers F1/R1 and F2/R2. **b** shows the PCR products generated by two pairs of primers F1/R1 (no GLI binding consensus sites) and F2/R2 (with one GLI binding consensus site) following chromatin immunoprecipitation with GLI1 (MYC) and GLI2 (Flag) antibodies (see Methods). Mouse IgG proteins were used as the negative control

To further determine whether *SOX2* expression is transcriptionally regulated by *GLI* molecules, we analyzed the promoter sequence of human *SOX2*, and discovered a *GLI*-consensus binding site (Fig. 8a). We performed ChIP assays in Colo357-GR and in BxPC3-GR cells following ectopic expression of GLI2 and GLI1 respectively. The genomic DNAs associated with GLI2 and GLI1 proteins were immunoprecipitated using specific tag antibodies, and the corresponding GLI binding DNA fragment was detected by PCR amplification with the flanking primers. We found that antibodies against ectopically expressed GLI1 and GLI2 proteins, but not the control IgG, were able to immunoprecipitate the DNA fragment containing the consensus GLI binding sequence (Fig. 8a, b). In contrast, an un-related DNA fragment was not detected in this assay (Fig. 8b). These results indicate that elevated *SOX2* expression in the resistant cells may be transcriptionally regulated by *GLI* molecules.

Taken all the data together, we found that acquired gemcitabine resistant pancreatic cancer cells have elevated expression of *GLI* transcription factors, either *GLI1* or *GLI2*, which is associated with elevated *SOX2* expression. We discovered that knockdown of *GLI* molecules (*GLI1* in BxPC3-GR, or *GLI2* in Colo357-GR) or *SOX2* sensitizes these cells to gemcitabine treatment. We have evidence to indicate that *SOX2* expression is regulated by GLI molecules, possibly through transcriptional regulation. The relevance of our findings to human cancer is reflected by the fact that high level of SOX2 protein expression is associated with a poor patient survival in a cohort of stage II pancreatic cancer patients following gemcitabine treatment. Thus, the disruption of the GLI-SOX2 signaling axis may be effective in sensitizing pancreatic cancer to gemcitabine-based therapy.

## Discussion

Our findings reveal an important mechanism underlying drug resistance in pancreatic cancer, one of the deadest cancer types. We showed that the Gli-Sox2 signaling axis is elevated in pancreatic cancer cells with acquired gemcitabine resistance. Down-regulation of *GLI1*, *GLI2* or *SOX2* sensitized pancreatic cancer cells to gemcitabine treatment. We have evidence to support that *SOX2* expression is regulated by GLI molecules, possibly through transcriptional regulation. The relevance of our studies was confirmed in pancreatic cancer specimens. We showed that high level of Sox2 expression is associated with poor patient survival in stage II disease following gemcitabine treatment. Thus, it is anticipated that strategies at disrupting the GLI-SOX2 signaling axis may be effective in sensitizing pancreatic cancer cells to gemcitabine-based therapy, the first line treatment for pancreatic cancer patients.

A previous report has shown activated hedgehog signaling involved in gemcitabine penetration. It was demonstrated that administration of smoothened antagonist IPI-926 and gemcitabine can significantly reduce metastases of pancreatic cancer in KPC mouse model [6]. The major mechanism for this effect is the fact that hedgehog signaling inhibition increases stromal penetration of gemcitabine. In comparison, our studies reveal a different mechanism by which acquired gemcitabine resistance may be contributed to the cancer intrinsic hedgehog signaling through elevated expression of GLI molecules, possibly through non-canonical regulation of GLI molecules. This theory is backed by our finding that inhibition of smoothened signaling is not sufficient to sensitize cancer cells to gemcitabine treatment in these pancreatic cancer cells (Fig. 3).

Our study is significant in a few aspects. First, our study indicates that the GLI-SOX2 signaling is both a biomarker for gemcitabine resistance and a target for future pancreatic cancer therapy. Second, strategies to suppress GLI functions may be effective in pancreatic cancer patients with relapsed disease following gemcitabine treatment. Our data also indicate that while expression of hedgehog ligands is elevated in the drug resistant cells, inhibition of ligand-dependent hedgehog signaling using smoothened antagonist BMS833923 was not effective in sensitizing pancreatic cancer cells to gemcitabine treatment. Although two smoothened antagonists are now approved by FDA for cancer treatment, they were not effective in improving gemcitabine-based treatment, as reported in several clinical trials [27, 28, 39–41]. Currently, there are no specific GLI inhibitors approved by FDA to suppress GLI activities. Several compounds have shown activities in reducing GLI signaling, including GANT61 [42] and Arsenic trioxide (ATO) [43]. ATO has been approved by FDA for treatment of acute promyelocytic leukemia [44–46]. Further studies are needed to test whether GLI1/2 inhibitors, including GANT61, are effective in reducing drug resistance in pancreatic cancer.

Furthermore, up-regulation of *SOX2* is commonly observed in other resistant cancer cells [47–49]. Resistance to chemotherapy and targeted therapies is a major issue both in the clinical care of cancer patients and in cancer research. For example, SOX2 is known to be responsible for resistance to anti-androgen based therapy in prostate cancer [49]. It remains unclear whether *SOX2* expression is regulated by GLI transcription factors under these conditions. Based on these results, agents specifically targeting SOX2 may be effective in cancer therapy.

In our tissue array analysis, we discovered an association between high SOX2 protein level with poor patient survival. Thus, it appears that patients with high Sox2 protein expression in the tumor may not suitable for gemcitabine treatment. This result should be confirmed by expression of *GLI1/2* molecules. However, there are no suitable GLI1/2 antibodies for immunohistochemistry, and these analyses cannot be performed in tissue arrays. Instead, we used TCGA data to determine whether high expression of *SOX2* and *GLI* molecules is associated with more cancer relapse following gemcitabine treatment. Through cbioportal (http://www.cbioportal.org/) analysis of 186 TCGA pancreatic cancer specimens, we found that patients with high expression of *GLI2* and *SOX2* in the tumor had ~70% chance of cancer relapse following gemcitabine treatment. In contrast, patients with low expression of *GLI2* and *SOX2* in the tumor had <60% of chance of cancer relapse. These results are consistent with our hypothesis that activation of the GLI-SOX2 signaling axis is an important factor for gemcitabine resistance in pancreatic cancer.

## Materials and methods

### Chemicals, antibodies and reagents

Gemcitabine was purchased from Besse Medical (West Chester, OH). BMS833923 is a potent inhibitor for smoothened signaling (EC50 = 50 nmol/L) [26]. BMS833923 was provided by Bristol–Myers Squibb [50–52]. Antibodies to GLI1 (Cat#2534, Cell Signaling Technology, Danvers, MA, USA) and SOX2 (Cat# 97959, Abcam, Cambridge, UK) were purchased commercially; Gli2 antibodies were provided by Dr. CC Hui [53]. Other antibodies include Myc-Tag (Cat# 9B11, Cell Signaling Technology, Danvers, MA, USA); Flag-Tag (Sigma, St. Luis, MO, USA); CD24 (clone ML5), histone H3 and IgG (BioLegend, San Diego, CA, USA).

Cell lines Colo357 and BxPC3 cells were obtained from the American Type Culture Collection (ATCC), and maintained according to the vendor’s instruction. In order to generate cell lines resistant to gemcitabine, Colo357 and BxPC3 cells were exposed to increasing concentrations of gemcitabine. Finally, Colo357-GR (gemcitabine resistance) and BxPC3-GR were maintained in the presence of 500 nM gemcitabine. All cell lines have been tested for their authenticity.

### Cell viability assay

Alamar Blue assay was used to determine cell viability [54–56]. 2000–4000 cells were seeded in each well of 96-well plates. Different amounts of gemcitabine were added or left untreated for specified amount of time. For cell viability, we added Alamar Blue (10 ul/well) for 2 h at 37 °C. Viable cells with active mitochondrial enzyme activity will generate fluorescence from Alamar Blue (Fisher Scientific), which can be measured by a plate reader (BioTek) (excitation 530 nm; emission 590 nm). IC50 values were generated from fluorescent reading using GraphPad Prism.

### RNA extraction, RT-PCR, and real-time PCR

We extracted total RNAs from cultured cells or tumorous tissues using Tri-RNA extraction reagent (Sigma, St. Luis, MO, USA), and performed real-time quantitative PCR analyses according to a previously published procedure [57]. For each sample, we used triplicates and used the comparative C_T_ (ΔΔCT) as described by the manufacturer (Applied Biosystems/ Fisher Scientific). The relative amount of target (2^−ΔΔCT^) was obtained by normalizing to an endogenous glyceraldehyde-3-phosphate dehydrogenase (*GAPDH*) and relative to a calibrator. All primers and probes were purchased from Applied Biosystems/Fisher Scientific). RT-PCR was used to detect possible existence of *GLI1* isoforms in the pancreatic cancer cell lines. Primers 5′-TGTTCAACTCGATGACCC-3′ and 5′-GTCATGGGGACCACAAGG-3′ were used to detect wild type *GLI1* (500 bp) and truncated *GLI1* (*tGLI1*) (377 bp). Primers 5′-GGCATCCGACAGAGGTGAGATGGAC-3′ and 5′-GAGCCCAGCGCCCAGACAGA-3′ or 5′-CTGTCTCAGGGAACCGTGGGTCTTTGT-3′ were used to detect full-length *GLI1* or *GLI1ΔN* in pancreatic cancer cell lines.

### ShRNA expression and plasmids

We purchased shRNAs specific to Gli2 or Gli1 from Sigma (St. Luis, Mo, USA) and forced their expression in cells through lentivirus-mediated expression. For every five shRNAs tested for each gene, we found at least two shRNAs with reduced expression of the target genes in this study. Plasmids for Gli1 and Gli2 expression were from our previous study [58] with a MYC (GLI1) or Flag (GLI2) tag at the N-terminus.

### Flow cytometry

We obtained single cells from cells or tissues, and subjected them to anti-CD24 antibody staining. Fluorescence labeled antibodies purchased from BioLegend (San Diego, CA, USA) were incubated with cells for 30 min on ice (with 1:200 dilutions).

### Orthotopic mouse model and subcutaneous xenografts of pancreatic cancer

Pancreatic cancer cells (Colo357, Colo357-GR-shNC and Colo357-GR-sh*GLI2*) with stable expression of luciferase were harvested in single cell suspension at a concentration of 1 × 10^6^ cells/ml. A total of 2 × 10^5^ cells (in 50 uL of growth medium) were injected into pancreas of the NOD/scid/ IL2Rgnull mice (NSG) with a 27-gauge needle as reported previously [59]. For subcutaneous xenografts, 1 × 10^6^ cells were injected subcutaneously into NSG mice. Tumors in subcutaneous models were measured with a caliper twice a week whereas the tumor in orthotopic mouse models was measured by luciferase activity within cancer cells once a week. Two to 3 weeks after tumor transplantation, the mice were weighed and randomized (with each group with similar tumor size, with less than 20% difference among the mice and <20% difference between groups) into two treatment groups: vehicle control or 25-mg/kg gemcitabine by i.v injection, twice per week for 2 weeks. Mice with no tumor growth will be excluded from further treatment study. Generally, we have at least three mice for each treatment group, and the experiment was carried out in both orthotopic and subcutaneous models. Mice were euthanized by carbon dioxide asphyxia after treatment. Tumor lesions in both models were harvested and divided into several portions. Some were snap-frozen in liquid nitrogen for mRNA extraction; some were embedded in paraffin; some were cut into small pieces with scissors and then digested with collagenase IV to obtain single-cell suspension for flow cytometry analysis. Animal studies were approved by Indiana University School of Medicine (IACUC# 11370).

### Immunohistochemistry staining

PDAC tissue microarray (TMA) was generated in IU School of Medicine by the Pancreatic Cancer Signature Center. The TMA contained 35 patients at IIB stage. All patients received gemcitabine chemotherapy. Overall survival was defined as the time interval between the date of histological diagnosis and the date of death from any cause. Immunohistochemistry was performed as described previously [60]. In brief, after removing paraffin and underwent a series of hydration steps, the slides were treated with endogenous peroxidase in 0.3% H_2_O_2_ for 30 min. After blocking non-specific binding sites with 1% BSA, tissue sections were incubated with specific SOX2 antibodies (at 1:1500 dilution) at 37 °C in humid chambers for 2 h. We detected the antibody binding with streptavidin–biotin–peroxidase complex/HRP (VECTOR, USA) and substrate 3, 3-diaminobenzidine for 3 min. Hematoxylin was used for counterstaining.

### ChIP assay

we performed ChIP assay according to a previously reported protocol [58]. Briefly, chromatin DNA was first cross-linked by 1% formaldehyde, and genomic DNAs sheared by sonication in the presence of protease inhibitors. After removing the pellets, we performed immunoprecipitation by incubation of the aqueous phase mixture with primary antibodies against histone H3, Myc, Flag or IgG (as a negative control). The precipitated DNA fragments were analyzed by PCR with the following *SOX2* promoter primers: Pair 1 F 5′-TGGTGCAGGGTACTTAAATGA-3′, pair 1 R GAGGACAGAGGTTTGGGTCT; Pair 2 F 5-GCGTCCCATCCTCATTTAAG-3′ and Pair2-R 5′-AGCAACAGGTCACACCACAC’3′. Please note that pair1 is for the GLI-binding consensus site-containing fragment whereas pair2 is for the fragment without such a site.

### Human specimens

Collection of human specimens were approved by The Institutional research board at Indiana University with IRB Study EX0909–22. All the patient information were removed before specimens were used by the end users in this study.

### Statistical analyses

Results are expressed as the mean ± SD. All statistical comparisons were made with a standard *t* test (two-tailed), using biostatistics software from GraphPad Prism. Sample size was determined by POWER analysis. We predict that the difference between the treatment group and the control group will be >50%. Using Chi-Square analysis, we will need 5 mice/ group to achieve a statistically significant data (80% POWER with type I error < 0.05). We have at least 6 mice (with 3 males and 3 females) in orthotopic and subcutaneous mouse models in our study. The criteria for significance were *p* < 0.05 for all comparisons.

## Electronic supplementary material

Supplementary Figures
