# Supplementary material for: The role of GLI-SOX2 signaling axis for gemcitabine resistance in pancreatic cancer
Source: Oncogene. 2018 Oct 31;38(10):1764–77. doi: 10.1038/s41388-018-0553-0 (PMC6408295; doi:10.1038/s41388-018-0553-0)
Supplement: Supplementary file 1 — Supplementary Figures [file 41388_2018_553_MOESM1_ESM.pdf]

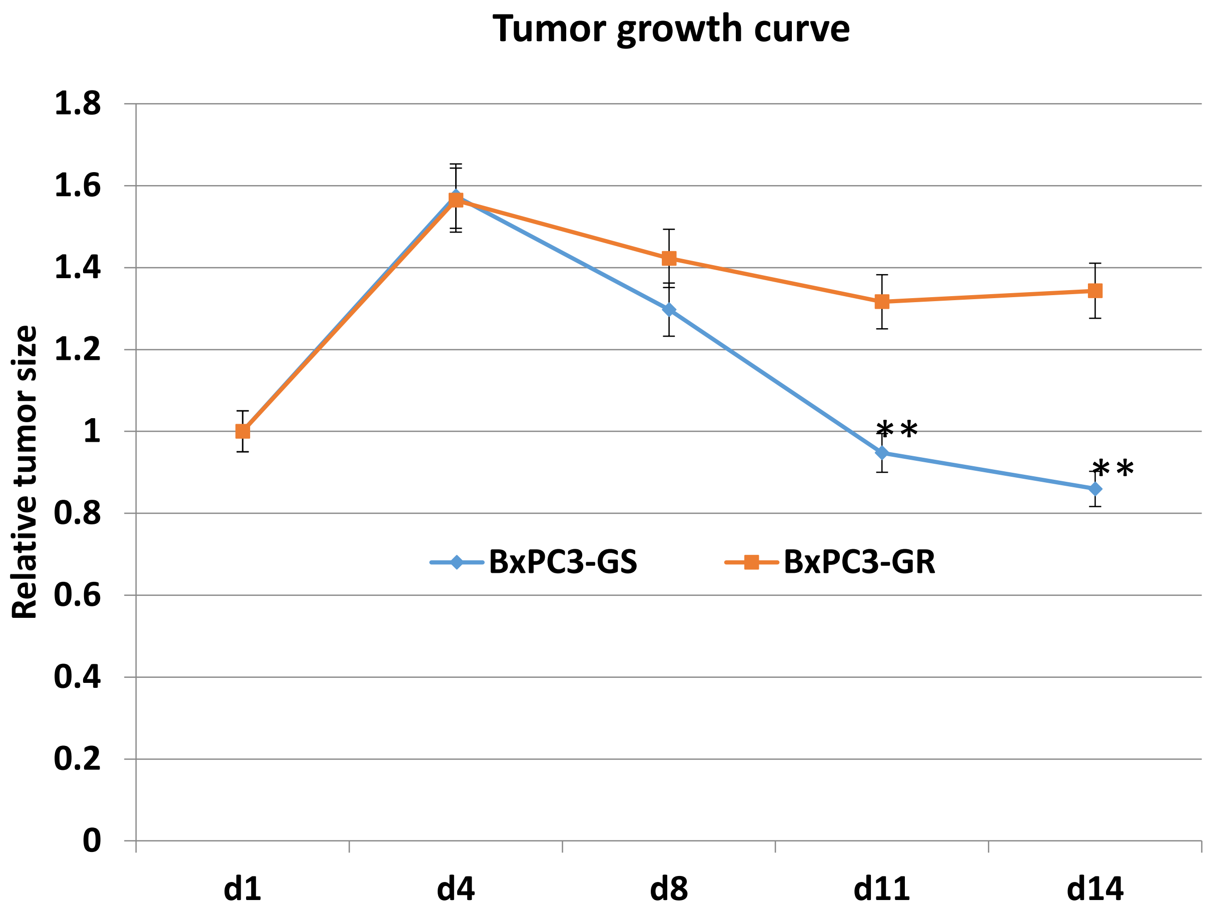


**Fig. S1- Response of BxPC3-derived tumors to gemcitabine in immune deficient NSG mice.** Tumors were formed following subcutaneous injection of BxPC3-GS and BxPC3-GR cells in NSG mice. Mice with tumors were treated with 25mg/kg gemcitabine by i.v injection (twice weekly), and tumor size was measured using a with a caliper twice a week. By day11, we observed significantly different responses to gemcitabine between gemcitabine resistant BxPC3-GR and gemcitabine sensitive BxPC3-GS cells (**p<0.05) , indicating that the tumors derived from BxPC3-GR are not sensitive to gemcitabine treatment.

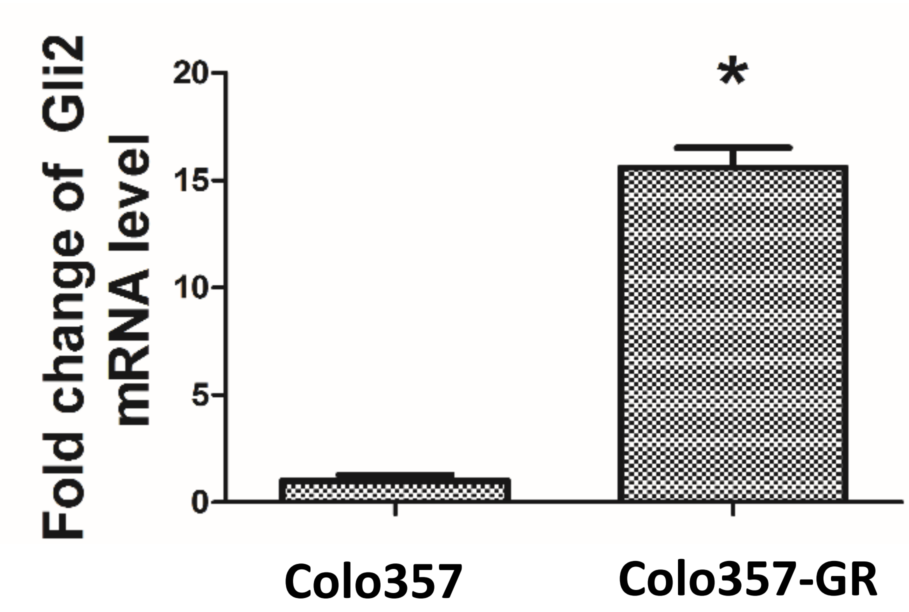


**Fig.S2. Detection of Gli2 in tumors formed from parental Colo357 (shown as Colo357) & gemcitabine resistant Colo357 (shown as Colo357-GR) by real-time PCR.** The results of the tumors are consistent with those from cultured cells (Fig.2B). * $p<0.005$

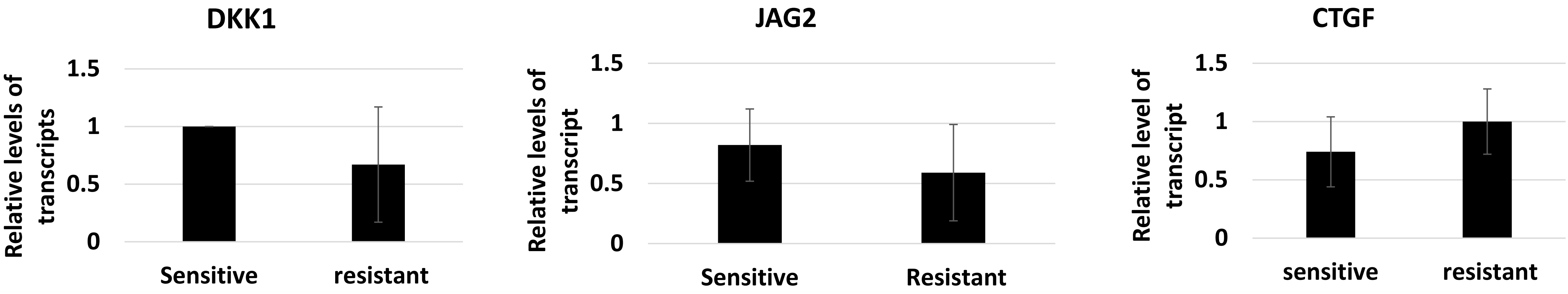


**Supplementary Figure 3- Expression of DKK1, JAG2 and CTGF in Colo357 cells** Total RNAs were extracted from gemcitabine resistant Colo357 cells and the parental Colo357 cells to determine expression of DKK1, JAG2 and CTGF by real-time PCR. Unlike Gli2, expression shown in Fig. 2B, no significant differences of expression in DKK1, JAG2 and CTGF were noted between Colo357 parental cells and gemcitabine resistant Colo357 cells, indicating that Gli2 expression is specifically up-regulated in the gemcitabine resistant cells. p values in all comparison >0.05.

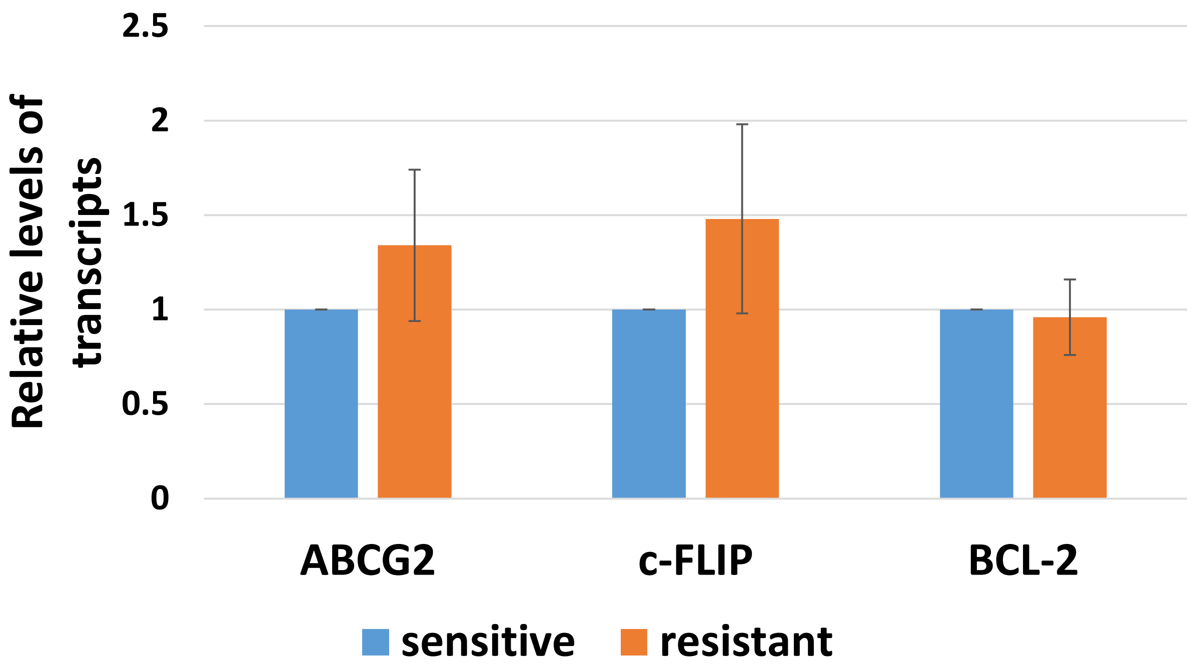


**Supplementary Figure 4. Expression of ABCG2, c-FLIP and BCL-2 in gemcitabine sensitive (as sensitive) and resistant (as resistant) Colo357 cells.** No significance was observed ($p>0.05$)

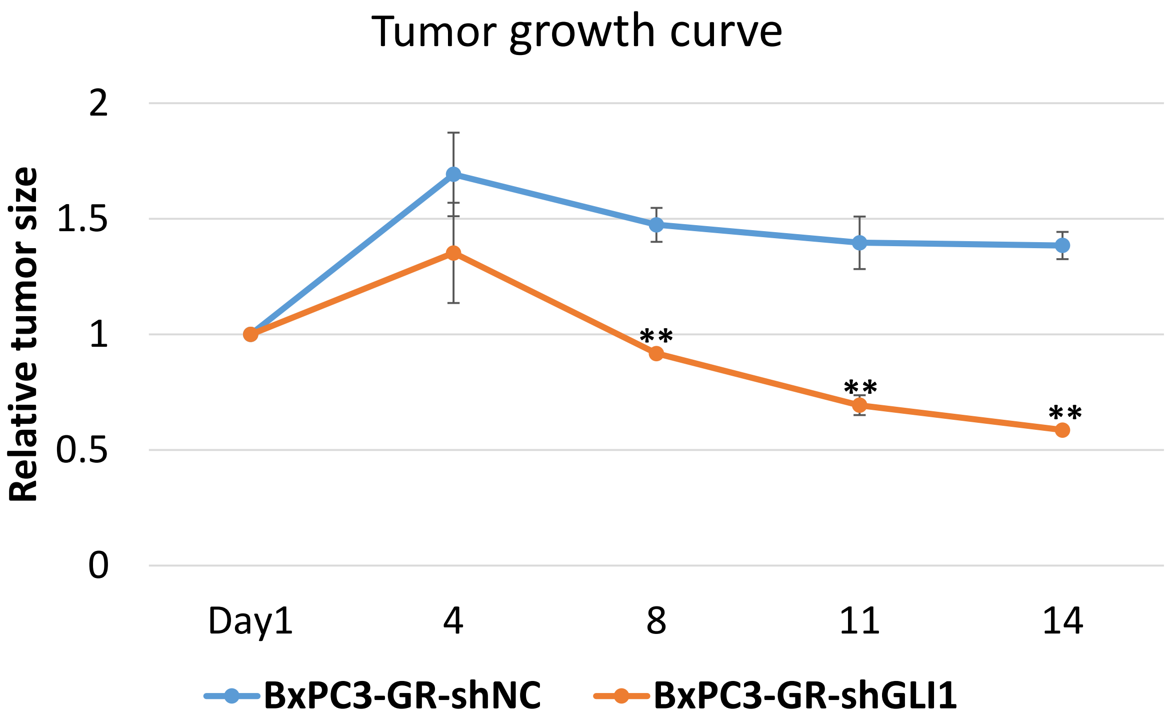


**Fig. S5- Down-regulation of GLI1 sensitizes BxPC3-GR-derived tumors to gemcitabine treatment.** Tumors were formed following subcutaneous injection of BxPC3-GR-shNC and BxPC3-GR-shGLI1 cells in NSG mice. Down-regulation of GLI1 was shown in Fig.3B. Mice with tumors were treated with 25mg/kg gemcitabine by i.v injection (twice weekly), and tumor size was measured using a with a caliper twice a week. From day 8, we started to observe significantly different responses to gemcitabine between BxPC3-GR–shNC and BxPC3-GR–shGLI1-derived tumors (**indicates $p<0.05$), indicating that GLI1 plays a role in gemcitabine response.
